# 3-(2,4-Dichloro­phen­yl)-2-oxo-1-oxaspiro­[4.5]dec-3-en-4-yl 2-methyl­prop-2-enoate

**DOI:** 10.1107/S1600536812016340

**Published:** 2012-04-25

**Authors:** Fan-rui Kong, Qiang Wang, Liang-zhong Xu

**Affiliations:** a College of Chemistry and Molecular Engineering, Qingdao University of Science and Technology, Qingdao 266042, People’s Republic of China

## Abstract

In the title mol­ecule, C_19_H_18_Cl_2_O_4_, the cyclo­hexane ring adopts a chair conformation. The furan ring is essentially planar and forms a dihedral angle of 82.1 (1)° with the benzene ring. In the crystal, weak C—H⋯O interactions are present.

## Related literature

For the potential biological activity of the title compound and the crystal structures of related compounds, see: Bretschneider *et al.* (2003 ▶). For the synthesis, see: Lu *et al.* (2008 ▶).
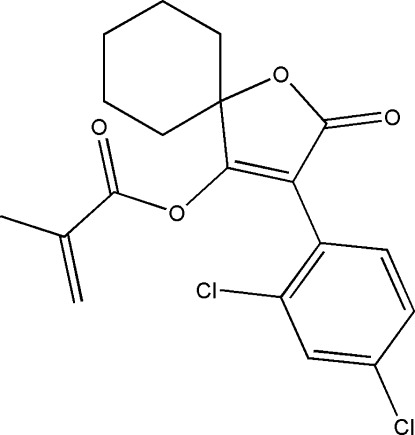


## Experimental

### 

#### Crystal data

C_19_H_18_Cl_2_O_4_
*M* *_r_* = 381.23
Monoclinic, 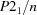

*a* = 10.759 (1) Å
*b* = 11.8778 (11) Å
*c* = 15.0130 (15) Å
β = 107.047 (4)°
*V* = 1834.3 (3) Å^3^
*Z* = 4
Mo *K*α radiation
μ = 0.37 mm^−1^
*T* = 113 K
0.22 × 0.20 × 0.14 mm

#### Data collection

Rigaku Saturn CCD diffractometer
Absorption correction: multi-scan (*CrystalClear*; Rigaku, 2005 ▶) *T*_min_ = 0.922, *T*_max_ = 0.950
17672 measured reflections
4371 independent reflections
3362 reflections with *I* > 2σ(*I*)
*R*_int_ = 0.034

#### Refinement

*R*[*F* ^2^ > 2σ(*F* ^2^)] = 0.035
*wR*(*F* ^2^) = 0.092
*S* = 1.03
4371 reflections
227 parameters
H-atom parameters constrained
Δρ_max_ = 0.55 e Å^−3^
Δρ_min_ = −0.36 e Å^−3^

### 

Data collection: *CrystalClear* (Rigaku, 2005 ▶); cell refinement: *CrystalClear*; data reduction: *CrystalClear*; program(s) used to solve structure: *SHELXS97* (Sheldrick, 2008 ▶); program(s) used to refine structure: *SHELXL97* (Sheldrick, 2008 ▶); molecular graphics: *SHELXTL* (Sheldrick, 2008 ▶); software used to prepare material for publication: *SHELXTL*.

## Supplementary Material

Crystal structure: contains datablock(s) I, global. DOI: 10.1107/S1600536812016340/lh5451sup1.cif

Structure factors: contains datablock(s) I. DOI: 10.1107/S1600536812016340/lh5451Isup2.hkl

Supplementary material file. DOI: 10.1107/S1600536812016340/lh5451Isup3.cml

Additional supplementary materials:  crystallographic information; 3D view; checkCIF report

## Figures and Tables

**Table 1:** Hydrogen-bond geometry (Å, °)

*D*—H⋯*A*	*D*—H	H⋯*A*	*D*⋯*A*	*D*—H⋯*A*
C11—H11⋯O1^i^	0.95	2.52	3.2470 (17)	133
C18—H18*B*⋯O1^ii^	0.95	2.56	3.4486 (17)	156

Symmetry codes: (i) 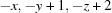; (ii) 
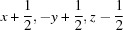
.

**Fig. 1.**
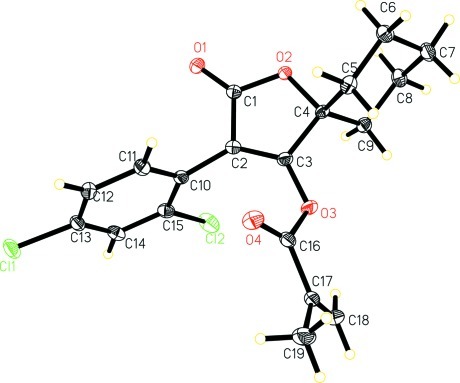
View of the title compound with displacement ellipsoids drawn at the 40% probability level.

## supplementary crystallographic information

### Comment

The title compound (I) was synthesized as a new compound with potential biological activity (Bretschneider *et al.*, 2003). We report herein its crystal structure.

In (I) (Fig. 1), all bond lengths and angles are normal and in a good agreement with those reported previously (Bretschneider *et al.*, 2003). The cyclohexane ring (C4—C9) adopts a chair conformation. The furan ring (O2/C1-C4) plane forms a dihedral angle of 82.1 (1)° with the benzene ring (C10—C15). In the crystal, weak intermolecular C—H···O hydrogen bonds are present.

### Experimental

The synthesis followed the prodedure of Lu *et al.* (2008). In a flask equipped with stirrer and reflux condenser, 3-(2,4-dichlorophenyl)-2-oxo-1-oxaspiro[4.5]dec-3-ene-4-ol 3.13 g (10.0 mmol), and triethylamine 5 ml was mixed in dichloromethane (30 ml), at 273-278K. The mixture was stirred, then methacryloyl chloride 1.25g (12.0 mmol) for was added dropwise for 1h, then the mixtures was left at room temperature for 3 h. The mixture was then washed with 1% HCl (60 ml) and water (60 ml), and the organic layer was dried over sodium sulfate. Excess dichloromethane was removed on a water vacuum pump to obtain an oily colorless product. The product was crystallized from methanol to afford the title compound 3.39 g (89% yield). Single crystals suitable for X-ray measurements were obtained from a solution of the title compound in acetone and methanol at room temperature.

### Refinement

H atoms were placed in calculated positions, with C—H = 0.95 - 0.99 Å, and included in the final cycles of refinement using a riding model, with *U*_iso_(H) = 1.2*U*_eq_(C) or 1.5*U*_eq_(C) for methyl C atoms.

### Crystal data

**Table:**

C_19_H_18_Cl_2_O_4_	*F*(000) = 792
*M**_r_* = 381.23	*D*_x_ = 1.381 Mg m^−^^3^
Monoclinic, *P*2_1_/*n*	Mo *K*α radiation, λ = 0.71073 Å
Hall symbol: -P 2yn	Cell parameters from 6104 reflections
*a* = 10.759 (1) Å	θ = 1.4–28.1°
*b* = 11.8778 (11) Å	µ = 0.37 mm^−^^1^
*c* = 15.0130 (15) Å	*T* = 113 K
β = 107.047 (4)°	Prism, colorless
*V* = 1834.3 (3) Å^3^	0.22 × 0.20 × 0.14 mm
*Z* = 4	

### Data collection

**Table:**

Rigaku Saturn CCD diffractometer	4371 independent reflections
Radiation source: rotating anode	3362 reflections with *I* > 2σ(*I*)
Multilayer monochromator	*R*_int_ = 0.034
Detector resolution: 14.63 pixels mm^-1^	θ_max_ = 27.9°, θ_min_ = 2.1°
ω and φ scans	*h* = −13→14
Absorption correction: multi-scan (*CrystalClear*; Rigaku, 2005)	*k* = −14→15
*T*_min_ = 0.922, *T*_max_ = 0.950	*l* = −19→19
17672 measured reflections	

### Refinement

**Table:**

Refinement on *F*^2^	Primary atom site location: structure-invariant direct methods
Least-squares matrix: full	Secondary atom site location: difference Fourier map
*R*[*F*^2^ > 2σ(*F*^2^)] = 0.035	Hydrogen site location: inferred from neighbouring sites
*wR*(*F*^2^) = 0.092	H-atom parameters constrained
*S* = 1.03	*w* = 1/[σ^2^(*F*_o_^2^) + (0.0557*P*)^2^] where *P* = (*F*_o_^2^ + 2*F*_c_^2^)/3
4371 reflections	(Δ/σ)_max_ = 0.002
227 parameters	Δρ_max_ = 0.55 e Å^−^^3^
0 restraints	Δρ_min_ = −0.36 e Å^−^^3^

### Special details

**Table:**

Geometry. All e.s.d.'s (except the e.s.d. in the dihedral angle between two l.s. planes) are estimated using the full covariance matrix. The cell e.s.d.'s are taken into account individually in the estimation of e.s.d.'s in distances, angles and torsion angles; correlations between e.s.d.'s in cell parameters are only used when they are defined by crystal symmetry. An approximate (isotropic) treatment of cell e.s.d.'s is used for estimating e.s.d.'s involving l.s. planes.
Refinement. Refinement of *F*^2^ against ALL reflections. The weighted *R*-factor *wR* and goodness of fit *S* are based on *F*^2^, conventional *R*-factors *R* are based on *F*, with *F* set to zero for negative *F*^2^. The threshold expression of *F*^2^ > σ(*F*^2^) is used only for calculating *R*-factors(gt) *etc*. and is not relevant to the choice of reflections for refinement. *R*-factors based on *F*^2^ are statistically about twice as large as those based on *F*, and *R*- factors based on ALL data will be even larger.

### Fractional atomic coordinates and isotropic or equivalent isotropic displacement parameters (Å^2^)

**Table:**

	*x*	*y*	*z*	*U*_iso_*/*U*_eq_	
Cl1	0.51046 (3)	0.62323 (3)	1.13695 (2)	0.02998 (11)	
Cl2	0.37184 (4)	0.19570 (3)	1.04198 (3)	0.03410 (12)	
O1	0.00843 (9)	0.29580 (7)	1.08091 (6)	0.0212 (2)	
O2	−0.07870 (8)	0.18353 (7)	0.95872 (6)	0.01666 (19)	
O3	0.10997 (9)	0.21926 (8)	0.80025 (7)	0.0243 (2)	
O4	0.10974 (10)	0.40685 (8)	0.77325 (7)	0.0279 (2)	
C1	0.00933 (12)	0.26065 (10)	1.00566 (9)	0.0162 (3)	
C2	0.09833 (12)	0.28979 (10)	0.95101 (9)	0.0174 (3)	
C3	0.05816 (13)	0.23083 (10)	0.87256 (9)	0.0182 (3)	
C4	−0.06139 (12)	0.16271 (10)	0.86751 (8)	0.0159 (3)	
C5	−0.18169 (13)	0.20806 (10)	0.79466 (9)	0.0202 (3)	
H5A	−0.1945	0.2881	0.8082	0.024*	
H5B	−0.1682	0.2040	0.7323	0.024*	
C6	−0.30301 (13)	0.14107 (11)	0.79411 (10)	0.0234 (3)	
H6A	−0.3215	0.1509	0.8545	0.028*	
H6B	−0.3785	0.1700	0.7443	0.028*	
C7	−0.28405 (14)	0.01632 (11)	0.77779 (10)	0.0252 (3)	
H7A	−0.3627	−0.0260	0.7794	0.030*	
H7B	−0.2720	0.0060	0.7154	0.030*	
C8	−0.16622 (14)	−0.02997 (11)	0.85182 (10)	0.0239 (3)	
H8A	−0.1534	−0.1099	0.8379	0.029*	
H8B	−0.1826	−0.0268	0.9133	0.029*	
C9	−0.04271 (13)	0.03628 (10)	0.85624 (9)	0.0194 (3)	
H9A	−0.0183	0.0230	0.7984	0.023*	
H9B	0.0293	0.0089	0.9094	0.023*	
C10	0.20514 (12)	0.37111 (10)	0.98884 (9)	0.0176 (3)	
C11	0.17792 (13)	0.48609 (11)	0.98462 (9)	0.0213 (3)	
H11	0.0932	0.5114	0.9514	0.026*	
C12	0.27197 (13)	0.56429 (11)	1.02792 (9)	0.0223 (3)	
H12	0.2523	0.6424	1.0241	0.027*	
C13	0.39456 (13)	0.52694 (11)	1.07668 (9)	0.0211 (3)	
C14	0.42685 (13)	0.41434 (11)	1.08054 (10)	0.0225 (3)	
H14	0.5123	0.3898	1.1127	0.027*	
C15	0.33101 (13)	0.33740 (11)	1.03600 (9)	0.0205 (3)	
C16	0.14479 (13)	0.31519 (11)	0.75941 (9)	0.0208 (3)	
C17	0.22568 (13)	0.28559 (12)	0.69809 (9)	0.0243 (3)	
C18	0.28787 (15)	0.18689 (13)	0.70703 (12)	0.0344 (4)	
H18A	0.2806	0.1346	0.7531	0.041*	
H18B	0.3394	0.1690	0.6673	0.041*	
C19	0.23706 (16)	0.37669 (14)	0.63240 (11)	0.0375 (4)	
H19A	0.2959	0.3523	0.5972	0.056*	
H19B	0.2716	0.4449	0.6677	0.056*	
H19C	0.1511	0.3926	0.5892	0.056*	

### Atomic displacement parameters (Å^2^)

**Table:**

	*U*^11^	*U*^22^	*U*^33^	*U*^12^	*U*^13^	*U*^23^
Cl1	0.02639 (19)	0.0348 (2)	0.02668 (19)	−0.01427 (15)	0.00461 (15)	−0.00520 (14)
Cl2	0.02374 (19)	0.02107 (19)	0.0554 (3)	0.00275 (13)	0.00842 (18)	0.00714 (15)
O1	0.0225 (5)	0.0261 (5)	0.0155 (5)	−0.0015 (4)	0.0066 (4)	−0.0027 (4)
O2	0.0173 (4)	0.0202 (4)	0.0135 (4)	−0.0023 (3)	0.0061 (4)	−0.0013 (3)
O3	0.0303 (5)	0.0248 (5)	0.0243 (5)	−0.0074 (4)	0.0182 (4)	−0.0054 (4)
O4	0.0303 (6)	0.0293 (5)	0.0268 (5)	0.0020 (4)	0.0127 (5)	0.0018 (4)
C1	0.0153 (6)	0.0157 (6)	0.0174 (6)	0.0022 (5)	0.0043 (5)	0.0021 (5)
C2	0.0163 (6)	0.0183 (6)	0.0184 (6)	0.0002 (5)	0.0064 (5)	0.0008 (5)
C3	0.0198 (6)	0.0181 (6)	0.0193 (6)	−0.0007 (5)	0.0100 (5)	−0.0001 (5)
C4	0.0184 (6)	0.0180 (6)	0.0131 (6)	−0.0013 (5)	0.0073 (5)	−0.0007 (5)
C5	0.0244 (7)	0.0189 (6)	0.0162 (6)	0.0027 (5)	0.0041 (5)	0.0018 (5)
C6	0.0173 (6)	0.0295 (7)	0.0205 (7)	0.0027 (5)	0.0010 (5)	0.0002 (5)
C7	0.0225 (7)	0.0275 (7)	0.0236 (7)	−0.0063 (6)	0.0036 (6)	−0.0028 (6)
C8	0.0260 (7)	0.0177 (6)	0.0268 (7)	−0.0038 (5)	0.0061 (6)	−0.0008 (5)
C9	0.0197 (6)	0.0177 (6)	0.0208 (7)	0.0011 (5)	0.0060 (5)	−0.0013 (5)
C10	0.0169 (6)	0.0216 (6)	0.0153 (6)	−0.0021 (5)	0.0064 (5)	−0.0009 (5)
C11	0.0185 (6)	0.0227 (7)	0.0218 (7)	−0.0004 (5)	0.0047 (5)	−0.0011 (5)
C12	0.0243 (7)	0.0199 (7)	0.0237 (7)	−0.0025 (5)	0.0085 (6)	−0.0032 (5)
C13	0.0204 (6)	0.0261 (7)	0.0172 (6)	−0.0087 (5)	0.0061 (5)	−0.0030 (5)
C14	0.0156 (6)	0.0283 (7)	0.0228 (7)	−0.0028 (5)	0.0044 (5)	0.0045 (5)
C15	0.0205 (7)	0.0204 (6)	0.0220 (7)	−0.0001 (5)	0.0087 (6)	0.0039 (5)
C16	0.0171 (6)	0.0276 (7)	0.0178 (6)	−0.0063 (5)	0.0051 (5)	−0.0015 (5)
C17	0.0203 (7)	0.0358 (8)	0.0185 (7)	−0.0114 (6)	0.0082 (6)	−0.0080 (6)
C18	0.0284 (8)	0.0465 (9)	0.0350 (9)	−0.0084 (7)	0.0195 (7)	−0.0147 (7)
C19	0.0334 (9)	0.0564 (10)	0.0272 (8)	−0.0130 (8)	0.0160 (7)	−0.0021 (7)

### Geometric parameters (Å, º)

**Table:**

Cl1—C13	1.7378 (13)	C8—C9	1.5291 (18)
Cl2—C15	1.7351 (13)	C8—H8A	0.9900
O1—C1	1.2071 (15)	C8—H8B	0.9900
O2—C1	1.3570 (15)	C9—H9A	0.9900
O2—C4	1.4562 (14)	C9—H9B	0.9900
O3—C3	1.3649 (15)	C10—C15	1.3904 (18)
O3—C16	1.3962 (16)	C10—C11	1.3943 (19)
O4—C16	1.1902 (16)	C11—C12	1.3870 (18)
C1—C2	1.4734 (17)	C11—H11	0.9500
C2—C3	1.3289 (18)	C12—C13	1.3807 (19)
C2—C10	1.4810 (17)	C12—H12	0.9500
C3—C4	1.5025 (17)	C13—C14	1.3789 (19)
C4—C5	1.5270 (18)	C14—C15	1.3933 (19)
C4—C9	1.5310 (17)	C14—H14	0.9500
C5—C6	1.5265 (18)	C16—C17	1.4827 (18)
C5—H5A	0.9900	C17—C18	1.337 (2)
C5—H5B	0.9900	C17—C19	1.493 (2)
C6—C7	1.5253 (19)	C18—H18A	0.9500
C6—H6A	0.9900	C18—H18B	0.9500
C6—H6B	0.9900	C19—H19A	0.9800
C7—C8	1.523 (2)	C19—H19B	0.9800
C7—H7A	0.9900	C19—H19C	0.9800
C7—H7B	0.9900		
			
C1—O2—C4	109.93 (9)	H8A—C8—H8B	107.9
C3—O3—C16	119.46 (10)	C8—C9—C4	111.66 (10)
O1—C1—O2	121.58 (11)	C8—C9—H9A	109.3
O1—C1—C2	128.67 (12)	C4—C9—H9A	109.3
O2—C1—C2	109.75 (10)	C8—C9—H9B	109.3
C3—C2—C1	105.91 (11)	C4—C9—H9B	109.3
C3—C2—C10	134.25 (12)	H9A—C9—H9B	108.0
C1—C2—C10	119.83 (11)	C15—C10—C11	117.74 (12)
C2—C3—O3	130.95 (12)	C15—C10—C2	122.54 (11)
C2—C3—C4	112.32 (11)	C11—C10—C2	119.49 (12)
O3—C3—C4	116.67 (11)	C12—C11—C10	121.34 (13)
O2—C4—C3	101.81 (10)	C12—C11—H11	119.3
O2—C4—C5	107.36 (10)	C10—C11—H11	119.3
C3—C4—C5	112.30 (10)	C13—C12—C11	119.05 (12)
O2—C4—C9	109.13 (10)	C13—C12—H12	120.5
C3—C4—C9	113.30 (10)	C11—C12—H12	120.5
C5—C4—C9	112.21 (10)	C14—C13—C12	121.58 (12)
C6—C5—C4	111.34 (10)	C14—C13—Cl1	118.88 (11)
C6—C5—H5A	109.4	C12—C13—Cl1	119.52 (10)
C4—C5—H5A	109.4	C13—C14—C15	118.31 (13)
C6—C5—H5B	109.4	C13—C14—H14	120.8
C4—C5—H5B	109.4	C15—C14—H14	120.8
H5A—C5—H5B	108.0	C10—C15—C14	121.92 (12)
C7—C6—C5	110.66 (11)	C10—C15—Cl2	119.98 (10)
C7—C6—H6A	109.5	C14—C15—Cl2	118.09 (10)
C5—C6—H6A	109.5	O4—C16—O3	122.02 (12)
C7—C6—H6B	109.5	O4—C16—C17	126.80 (12)
C5—C6—H6B	109.5	O3—C16—C17	111.16 (11)
H6A—C6—H6B	108.1	C18—C17—C16	120.88 (13)
C8—C7—C6	110.82 (11)	C18—C17—C19	124.49 (13)
C8—C7—H7A	109.5	C16—C17—C19	114.52 (13)
C6—C7—H7A	109.5	C17—C18—H18A	120.0
C8—C7—H7B	109.5	C17—C18—H18B	120.0
C6—C7—H7B	109.5	H18A—C18—H18B	120.0
H7A—C7—H7B	108.1	C17—C19—H19A	109.5
C7—C8—C9	111.89 (11)	C17—C19—H19B	109.5
C7—C8—H8A	109.2	H19A—C19—H19B	109.5
C9—C8—H8A	109.2	C17—C19—H19C	109.5
C7—C8—H8B	109.2	H19A—C19—H19C	109.5
C9—C8—H8B	109.2	H19B—C19—H19C	109.5
			
C4—O2—C1—O1	175.71 (11)	C7—C8—C9—C4	52.98 (15)
C4—O2—C1—C2	−4.42 (13)	O2—C4—C9—C8	67.09 (14)
O1—C1—C2—C3	−178.77 (13)	C3—C4—C9—C8	179.75 (11)
O2—C1—C2—C3	1.38 (14)	C5—C4—C9—C8	−51.77 (14)
O1—C1—C2—C10	1.1 (2)	C3—C2—C10—C15	−84.2 (2)
O2—C1—C2—C10	−178.79 (10)	C1—C2—C10—C15	95.97 (15)
C1—C2—C3—O3	−174.75 (13)	C3—C2—C10—C11	101.37 (18)
C10—C2—C3—O3	5.4 (3)	C1—C2—C10—C11	−78.41 (16)
C1—C2—C3—C4	2.16 (15)	C15—C10—C11—C12	−1.63 (19)
C10—C2—C3—C4	−177.64 (13)	C2—C10—C11—C12	173.02 (12)
C16—O3—C3—C2	−50.0 (2)	C10—C11—C12—C13	−0.5 (2)
C16—O3—C3—C4	133.19 (12)	C11—C12—C13—C14	2.3 (2)
C1—O2—C4—C3	5.30 (12)	C11—C12—C13—Cl1	−176.24 (10)
C1—O2—C4—C5	−112.83 (10)	C12—C13—C14—C15	−1.9 (2)
C1—O2—C4—C9	125.32 (10)	Cl1—C13—C14—C15	176.66 (10)
C2—C3—C4—O2	−4.59 (14)	C11—C10—C15—C14	2.05 (19)
O3—C3—C4—O2	172.81 (10)	C2—C10—C15—C14	−172.42 (12)
C2—C3—C4—C5	109.94 (13)	C11—C10—C15—Cl2	−178.86 (10)
O3—C3—C4—C5	−72.66 (14)	C2—C10—C15—Cl2	6.66 (18)
C2—C3—C4—C9	−121.62 (12)	C13—C14—C15—C10	−0.34 (19)
O3—C3—C4—C9	55.77 (15)	C13—C14—C15—Cl2	−179.44 (10)
O2—C4—C5—C6	−66.13 (13)	C3—O3—C16—O4	−14.1 (2)
C3—C4—C5—C6	−177.23 (10)	C3—O3—C16—C17	166.98 (11)
C9—C4—C5—C6	53.77 (14)	O4—C16—C17—C18	161.57 (15)
C4—C5—C6—C7	−56.48 (15)	O3—C16—C17—C18	−19.56 (18)
C5—C6—C7—C8	57.59 (15)	O4—C16—C17—C19	−14.7 (2)
C6—C7—C8—C9	−56.08 (15)	O3—C16—C17—C19	164.16 (12)

### Hydrogen-bond geometry (Å, º)

**Table:**

*D*—H···*A*	*D*—H	H···*A*	*D*···*A*	*D*—H···*A*
C11—H11···O1^i^	0.95	2.52	3.2470 (17)	133
C18—H18*B*···O1^ii^	0.95	2.56	3.4486 (17)	156

Symmetry codes: (i) −*x*, −*y*+1, −*z*+2; (ii) *x*+1/2, −*y*+1/2, *z*−1/2.
